# Early Transcatheter Heart Valve Pannus Leading to Coronary Obstruction Managed With Orthotopic Chimney Stenting

**DOI:** 10.1016/j.cjco.2022.01.008

**Published:** 2022-02-01

**Authors:** Mariama Akodad, Anthony Chuang, Abdul Ihdayhid, Andrew G. Chatfield, Jonathon Leipsic, Anson Cheung, David A. Wood, Anthony Della Siega, M. Bilal Iqbal, John G. Webb, Janarthanan Sathananthan

**Affiliations:** a Centre for Heart Valve Innovation, Centre for Cardiovascular Innovation, St. Paul’s Hospital, University of British Columbia, Vancouver, British Columbia, Canada; b Royal Jubilee Hospital, Victoria, British Columbia, Canada

A 78-year-old woman was admitted with acute coronary syndrome 6 months after transcatheter aortic valve replacement (TAVR) with a 26-mm Evolut R transcatheter heart valve (THV; Medtronic, Minneapolis, MN). Coronary angiography was challenging, and only nonselective images were obtained. She was treated with dual antiplatelet therapy and therapeutic anticoagulation. However, despite aggressive antithrombotic management for 1 week, she continued to deteriorate and was eventually referred to our centre with refractory angina and rising troponin up to 2432 ng/L (normal: < 14 ng/L).

An urgent cardiac computed tomography (CT) scan showed extensive pannus on the THV frame, expanding up to the level of the coronary arteries and the THV commissure posts positioned in front of both coronary artery ostia (Fig. 1, A and B). Thrombus was also highlighted in the sinuses of Valsalva, as a consequence of impaired flow caused by the tissue ingrowth. The patient was deemed to be at prohibitive risk for surgery, due to a porcelain aorta. Considering the critical presentation, with worsening symptoms and dynamic electrocardiogram changes in the anterior leads, she was referred for percutaneous coronary intervention to improve left main perfusion.

**Figure 1:**
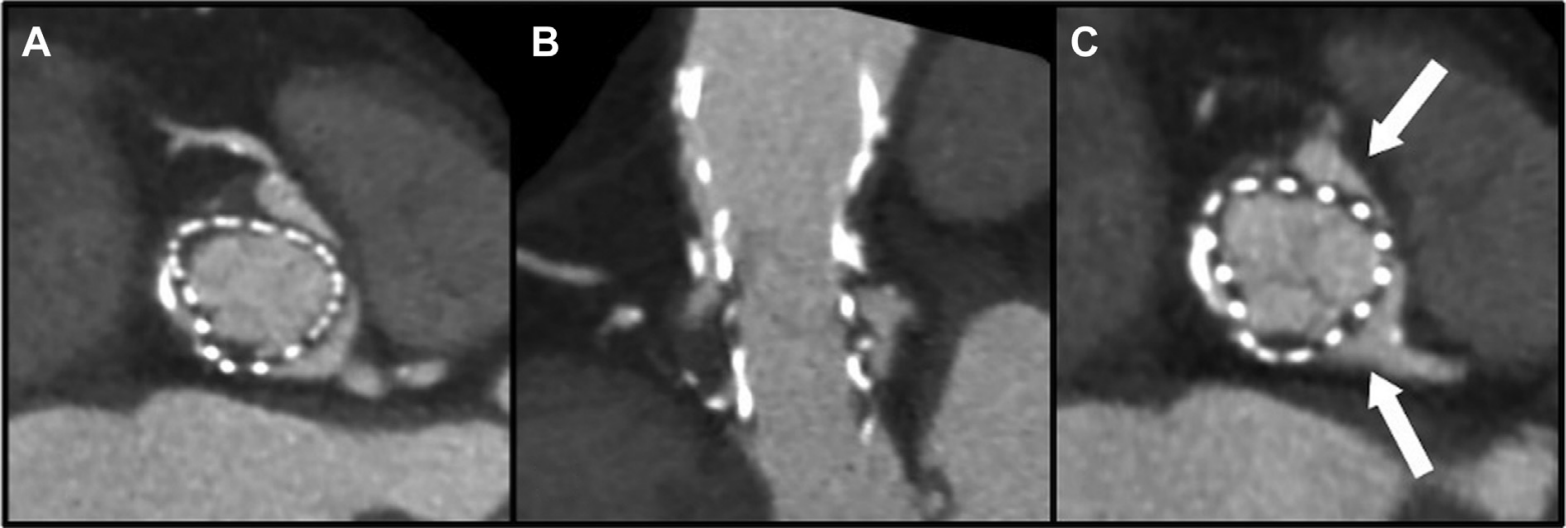
(**A, B**) Thrombus and pannus formation on the Evolut R transcatheter heart valve (Medtronic, Minneapolis, MN). (**C**) The transcatheter heart valve neo-commissure in front of the coronary ostia (**white arrows**).

A 6 Fr Judkins Left (Cordis, FL) 3.0 guiding catheter was unable to cross the THV struts, with widespread ST depression on the electrocardiogram, while the guiding catheter was interacting with the THV frame (Fig. 2A). After wiring the left anterior descending artery, a Guideliner (Vascular Solutions, Morrisville, NC) was advanced over a 2.5 x 12-mm balloon (Fig. 2B; Video 1 , view video online). A first 3.5 x 28-mm drug-eluting stent was implanted from the left anterior descending artery back across the THV strut (Fig. 2C; Video 2 , view video online). Despite stent reinflation at high pressure, a significant waist at the level of the THV strut was noted, and the decision was made to implant a second overlapping stent to improve radial strength at this level. Intravascular imaging was not performed, as the stent under expansion was visible angiographically, and to avoid any potential for stent distortion. A second overlapping 4 x 12-mm stent then was inserted from the left main carina back into the THV frame, to improve radial strength, and post-dilated with a 4.5-mm noncompliant balloon with an excellent angiographic result and immediate resolution of chest pain and ST depression (Fig. 2D; Videos 3 and 4, , view videos online). Given the complete resolution of symptoms and right coronary artery small diameter, this was managed medically. The patient was discharged under oral anticoagulation (rivaroxaban) and clopidogrel. Follow-up with alternated CT scan and stress test was recommended. At 6-month follow-up, the patient was asymptomatic, and a CT scan confirmed stent patency.

**Figure 2:**
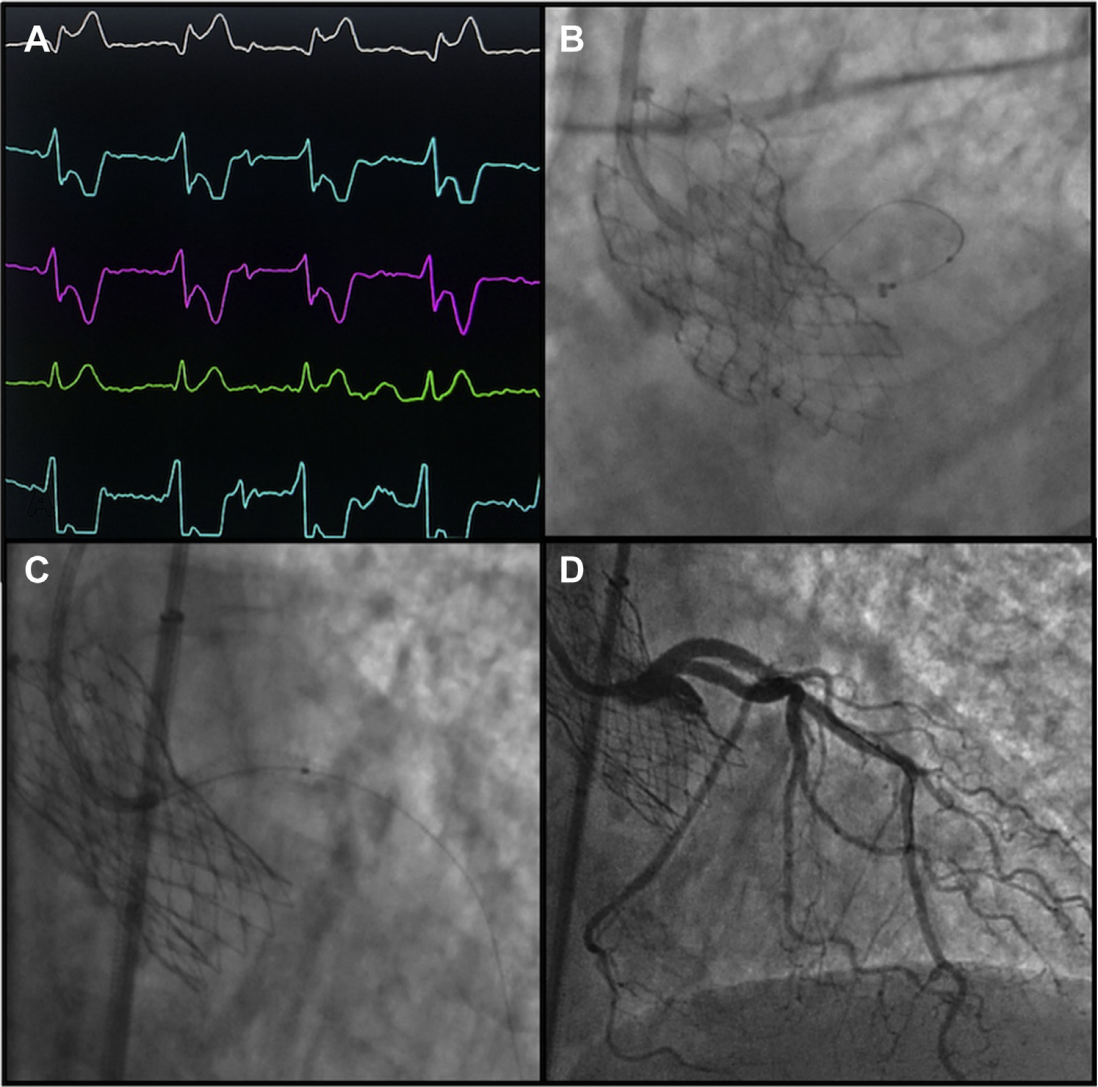
(**A**) Electrocardiogram changes during catheter interaction with the Evolut R transcatheter heart valve (Medtronic, Minneapolis, MN). (**B**) Guideliner (Vascular Solutions, Morrisville, NC) advanced into left main artery over coronary balloon. (**C**) Implantation of first stent. (**D**) Final angiographic result.

Coronary access may be challenging after TAVR, with 16% of commissural misalignment reported with the Evolut R THV.^1^^,^^2^ Commissural alignment may be improved with the Evolut platform, according to recent recommendations, including the positioning of the flush port at the “3 o’clock” position and the use of the cusp-overlap view for THV positioning.^3^ The CT scan may be useful to define the mechanism of post-TAVR angina and to guide subsequent coronary intervention.^1^^,^^2^ The orthotopic chimney-stenting, allowing coronary stenting through the THV struts, has been described previously and may be facilitated using a shorter guiding catheter and a Guideliner.^4^^,^^5^

**Novel Teaching Points**

• CT scanning is key to guiding coronary access and understanding the mechanism of acute coronary syndrome in patients with previous TAVR.
• Guideliner may be of help in facilitating coronary access and stent delivery through the THV struts.
• Orthotopic chimney stenting may be an acceptable alternative to surgery in cases with coronary access impairment and prohibitive surgical risk.
• A double layer of stent may improve radial strength at the THV strut level.

## Supplementary Material

Video 1 Guideliner (Vascular solution, NC) was advanced over a 2.5 **×** 12-mm balloon in the LAD.

Video 2 Implantation of a first 3.5 **×** 28-mm drug-eluting stent from the left anterior descending artery back across the THV strut.

Video 3 Implantation of a second overlapping 4 **×** 12-mm stent from the left main carina back into the THV frame.

Video 4 Final angiographic result showing left main, LAD and circumflex patency.
